# The age- and sex-specific occurrence of bothersome neck pain in the general population – results from the Stockholm public health cohort

**DOI:** 10.1186/1471-2474-13-185

**Published:** 2012-09-24

**Authors:** Eva Skillgate, Cecilia Magnusson, Michael Lundberg, Johan Hallqvist

**Affiliations:** 1 Institute of Environmental Medicine, Karolinska Institutet, Box 210, Stockholm, SE-17177, Sweden; 2 Scandinavian College of Naprapathic Manual Medicine, Kräftriket 23A, Stockholm, SE-11419, Sweden; 3 Department of Public Health Sciences, Division of Public Health Epidemiology, Karolinska Institutet, Norrbacka, Stockholm, SE-17176, Sweden; 4 Department of Public Health and Caring Sciences, Preventive Medicine, Uppsala University, Box 564, Uppsala, SE-75122, Sweden

## Abstract

**Background:**

Neck pain is very common but the occurrence of bothersome neck pain is not well described. Therefore our objective was to report on the prevalence and incidence of, as well as the rate of recovery from, bothersome neck pain in men and women of different ages in the general population.

**Methods:**

We used data from a recently conducted population-based cohort study, comprising 23,794 individuals in Stockholm County, Sweden. Study participants were surveyed with a self-administered questionnaire in 2002/2003 and 2007, and information on episodes of neck pain was gathered at baseline and at follow-up. We then measured bothersome neck pain in 2005 and 2006 retrospectively in 2007 using the follow-up questionnaire.

**Results:**

The one-year prevalence of bothersome neck pain for at least seven consecutive days was 25% (95% confidence interval (CI): 24–25) among women and 16% (95% CI: 15–16) among men, peaking in individuals aged 30–59 years. The one-year incidence proportion of bothersome neck pain was 7% (95% CI: 6–7) among women, and 4% (95% CI: 4–5) among men. Women recovered more infrequently than men. The one-year incidence proportion of recovery (of at least one year duration) was 11% (95% CI: 10–12) among women and 14% (95% CI: 12–16) among men.

**Conclusion:**

Bothersome neck pain is most common in middle-aged individuals. Women are more likely than men to have and to develop bothersome neck pain, and less likely to recover from such pain. Younger men and women have a higher incidence, but recover more often from bothersome neck pain than older individuals.

## Background

Neck pain (NP) is a common disorder in the general population but the age and sex-specific occurrence of bothersome NP is not well described. According to recent systematic reviews, the one-year prevalence of NP of any type varies between 2% and 80% [1-3], and is higher among women than men [1,3,4]. The one-year prevalence of persistent or regularly recurrent NP (> 3 months) in Sweden is 22.9% among women and 14.5% among men [5]. Although NP has been reported to be common among centenarians [6], it remains unclear whether the occurrence increases with age [3]. One survey found that prevalence peaked among middle-aged individuals [7], whereas another found an equally high prevalence among 31–50 year-olds as among 51–70 year-olds [8].

Knowledge about the age- and sex-specific incidence proportion of NP is even more inadequate. In the United Kingdom, the one-year cumulative incidence in the general population was 17.9% for NP that lasted for >1 day [9], without statistically significant fluctuations across age groups (ranging from 18 to 74 years). In the general population in Saskatchewan, Canada, the annual incidence of disabling NP was 14.6% [10]. We have not found any sex-specific information on incidence of bothersome NP.

Regarding the course of NP in the general population, a systematic review indicated that 50-75% of individuals with current NP report NP again within 1 to 5 years [11]. Coté et al. reported an annual overall recovery rate of 36.6%, albeit lower among women [10]. We have not found any age-specific information on the recovery rate from bothersome NP.

In summary there is insufficient knowledge regarding the age- and sex-specific occurrence and course of bothersome NP. Such knowledge is important for the appraisal of the burden of disease and for the planning of preventive strategies and other health services. Therefore our objective was to report on the age- and sex-specific a) one-year prevalence of bothersome NP (BNP), b) incidence proportion of the onset of at least one episode of BNP among individuals free from such pain during the preceding year, and c) recovery measured as the incidence proportion of not having BNP among individuals with such pain the preceding year. Our sample comprised the general population of Stockholm County, Sweden.

## Methods

This study was carried out in compliance with the Helsinki Declaration, and was approved by the Regional Ethical Review Board in Stockholm, Sweden (2007/545-31 and 2009/457-31). Written informed consent, included in the questionnaires, was obtained from each participant.

### Study population and design

**Figure 1:**
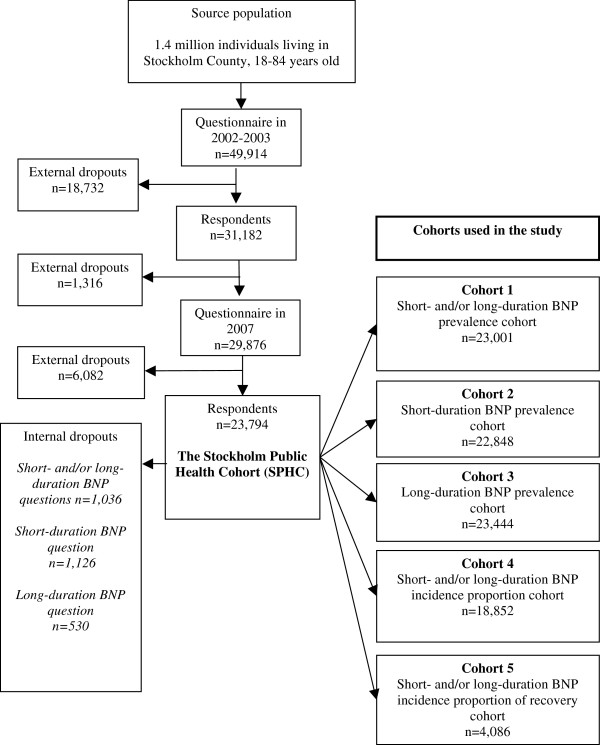
Flow chart describing the selection of study participants in the cohorts.

The Stockholm Public Health Cohort is a recently established prospective study, set within the framework of the Stockholm County Council Public Health Surveys with the aim of collecting data for regional public and occupational health surveillance in the general population [12]. Between October 29^th^ in 2002 and March 22^nd^ in 2003, 49,914 individuals (age 18–84 years) were randomly selected after stratification for municipality from 1.4 million eligible residents in Stockholm County (the source population), and sent an extensive postal questionnaire (Figure 1). Responders (n = 31,182; 62%) were resurveyed in 2007 via an extensive mixed mode postal/web-based questionnaire. Participants in both surveys (n = 23,794, corresponding to a 76% retention rate) constitute the study population used for the analyses in this study. A comparison between the study population in this study and Stockholm County census data shows that men, those under the age of 45, those born outside Sweden, and those unemployed or in the lowest quartile of income, were more likely to be non-responders to the surveys (unpublished data). To describe the characteristics of the participants in the cohort, we used information from the baseline questionnaire in 2002/2003. To describe the occurrence of BNP, retrospective information from the follow-up questionnaire in 2007 was used. The most valid information on the occurrence of BNP was judged to be from 2006 since that was the last full year before the questionnaire was answered in 2007. We used information from 2005 to study the incidence proportion.

### Definitions of measurements and cohorts

We measured BNP in 2005 and 2006 using retrospective questions in the follow-up questionnaire sent out in 2007. The exact wording of the questions was: 1) “During the last five-year period, have you had neck pain for at least three consecutive months that has bothered you considerably?”. 2) “If yes, which year/years did you have such pain? a. 2002, b. 2003, c. 2004, d. 2005, e. 2006, f. 2007”. 3) “During the last five-year period, have you had neck pain, for at least seven consecutive days but less than three consecutive months, that has bothered you considerably?” 4) “If yes, which year/years did you have such pain? a. 2002, b. 2003, c. 2004, d. 2005, e. 2006, f. 2007”. The full questionnaire used in the survey is attached in the Appendix 1.

Our case definitions are accordingly *survey-based bothersome neck pain (BNP) of short or long duration in the general population*. This is based on the classification proposed by the Bone and Joint Decade 2000–2010 Task Force on Neck Pain regarding Axis I (source of subjects), Axis II (setting and sampling) and Axis IV (duration) [13]. To describe the duration of the NP we used the terminology *short duration* (at least seven consecutive days but less than three consecutive months) or *long duration* (at least three consecutive months) as suggested by the Bone and Joint Decade 2000–2010 Task Force on Neck Pain and Its Associated Disorders [13,14]. To describe the severity of the NP (Axis III) we used the word bothersome in the questionnaire, which has been shown to be valid to measure severity of low back pain, and to identify people in the highest category of low back pain and disability [15].

We defined five sub-cohorts of the Stockholm Public Health Cohort for the analyses in this study, based on the retrospective questions in the follow-up questionnaire described above.

*Cohort 1*, the “short- and/or long-duration BNP prevalence cohort” (n = 23,001): those who answered both BNP questions in the full cohort sample.

*Cohort 2*, the “short-duration BNP prevalence cohort“ (n = 22,848): those who answered the short-duration BNP questions.

*Cohort 3*, the “long-duration BNP prevalence cohort“ (n = 23,444): those who answered the long-duration BNP questions.

*Cohort 4*, the “short- and/or long-duration BNP incidence proportion cohort” (n = 18,852): those who answered both the BNP questions and reported no such NP in 2005.

*Cohort 5*, the “short- and/or long-duration BNP incidence proportion of recovery cohort” (n = 4,086): those who answered both the BNP questions and reported short-and/or long-duration BNP in 2005.

How we selected study participants in the cohort is also described in Figure 1.

### Statistical analysis

We considered the sample of the Stockholm County population used in this study to be an actual random sample, since weighted analyses according to the stratified sampling scheme showed only negligible differences in the results.

Figure 1 and Table 1 provide a detailed description of the disorder estimates (the prevalence, the incidence proportion and the incidence proportion of recovery) and give the cohorts used for the analyses.

**Table 1:** Definitions of outcomes, sample size and status at baseline and follow-ups in the study

**Outcome**	**Sample at risk**^**a**^**, n**	**Baseline**	**Follow-up**
One-year prevalence of short-duration BNP in 2006	10,083 men and 12,765 women	The proportion of individuals in the cohort having experienced at least one episode of BNP in at least 7 consecutive days, but less than 3 consecutive months in 2006 according to a retrospective question in the follow-up questionnaire in 2007	-
One-year prevalence of long-duration BNP in 2006	10,298 men and 13,146 women	The proportion of individuals in the cohort having experienced constant or at least one episode of bothersome neck pain in at least 3 consecutive months in 2006 according to a retrospective question in the follow-up questionnaire in 2007	-
One-year prevalence of short- and/or long-duration BNP in 2006	10,123 men and 12,878 women	The proportion of individuals in the cohort having experienced at least one episode of short-duration BNP and/or long-duration BNP in 2006 according to a retrospective question in the follow-up questionnaire in 2007	-
Incidence proportion of short- and/or long-duration BNP in 2006	8,747 men and 10,105 women	Individuals without short or long- duration BNP in 2005 according to a retrospective question in the follow-up questionnaire in 2007.	The proportion of episodes (1-2 episodes per individual) in the cohort of short- and/or long-duration BNP in 2006 according to a retrospective question in the follow-up questionnaire in 2007
Incidence proportion of recovery from short- and/or long-duration BNP in 2006	1,358 men and 2,728 women	Individuals with short- and/or long-duration BNP in 2005 according to a retrospective question in the follow-up questionnaire in 2007	The proportion of individuals without short- or long-duration BNP in 2006 according to a retrospective question in the follow-up questionnaire in 2007

^a^Those in the cohorts that have answered the different questions about neck pain.

#### Prevalence

The sex-specific one-year prevalence of *short- and/or long-duration BNP* in 2006 was estimated together with corresponding 95% confidence intervals (95% CI), for all and for six age groups (18–29, 30–39, 40–49, 50–59, 60–69 and 70–84 years at baseline). That is equal to the number of cases divided by the number of study persons in the cohorts as well as in each age strata, separately for men and women. Hence the prevalence is the proportion of persons having reported that they had NP anytime during 2006, sometimes called the period prevalence [16]. Table 1 gives more detailed information about these calculations.

#### Incidence proportion

The one-year incidence proportion of developing *short- and/or long-duration BNP* with corresponding 95% CI was estimated as follows: Within a cohort of individuals free from *short- and long-duration BNP* in 2005, the number of cases with *short- and/or long-duration BNP* in 2006 was divided by the number of study persons free from *short- and long-duration BNP* in 2005 in the cohort and in each age strata and separately for men and women.

Within a cohort of individuals with short- and/or long-duration BNP in 2005, the one-year incidence proportion of recovery with at least one year duration of no BNP with corresponding 95% CI was estimated as follows: The number of men and women respectively free from BNP in 2006 was divided by the number of men and women with short- and/or long-duration BNP in 2005 in the cohort and in each age strata. Table 1 gives more detailed information about how the incidences were calculated.

We conducted all the analyses using SAS version 9.1 (SAS Institute Inc).

## Results

Table 2 presents the characteristics of the participants in the Stockholm Public Health Cohort. The mean age of the participants was 48 years, 56% were women, 20% lived alone, 16% were born abroad and 20% were retired.

**Table 2:** Characteristics of the participants in the Stockholm Public Health Cohort, and in age sub-groups

	**All**	**Age 18–29**	**Age 30–39**	**Age 40–49**	**Age 50–59**	**Age 60–69**	**Age 70–84**
	**(n = 23,794)**	**(n = 3,374)**	**(n = 4,751)**	**(n = 4,276)**	**(n = 5,087)**	**(n = 3,639)**	**(n = 2,667)**
**Characteristics**							
Women, %	56	60	60	56	55	50	56
Age, mean (SD)	48 (16)	-	-	-	-	-	-
Living alone, %	20	20	15	12	18	25	39
No parent responsibility for children under the age of 25, %	57	83	33	19	53	88	92
Home and household work ≥ 3h/day, %	13	5	12	14	8	16	26
Born abroad (Immigrant), %	16	11	14	20	16	17	14
Unemployed, %	3	4	3	3	3	1	0
Retired, %	20	0	1	3	7	51	92
Student, %	6	30	5	2	0	0	0
Strained financial situation ^a^, %	14	33	19	16	8	5	3
Daily smoking, %	15	13	12	19	20	15	9
No alcohol the preceding year, %	10	9	8	8	8	11	19
Body Mass Index, mean (SD)	25 (4)	23 (4)	24 (4)	25 (5)	26 (4)	26 (4)	25 (4)
Sedentary leisure time ^b^, %	14	15	15	16	14	11	14
Regular exercise in leisure time ^c^, %	13	22	14	13	11	12	8
Psychological distress ^d^, %	21	33	28	23	19	11	11
Much stress at work ^e^, %	32	28	43	45	40	16	1
Bad general health, %	5	3	3	5	6	5	6
Pain from the lower back at least 2 days per week the preceding six months, %	17	11	12	16	19	20	26

^a^Obliged to borrow money from relatives or friends to pay the rent or food once or more often, during the preceding year.

^b^Walking, bicycling, etc. less than two hours per week.

^c^Running, swimming, tennis, badminton, workout etc. at least three times a week, and for at least 30 minutes each time

^d^GHQ-12 score ≥3.

^e^No time to talk or even think of anything else but work due to stressful work situation, in 50% of the working time or more.

**Figure 2:**
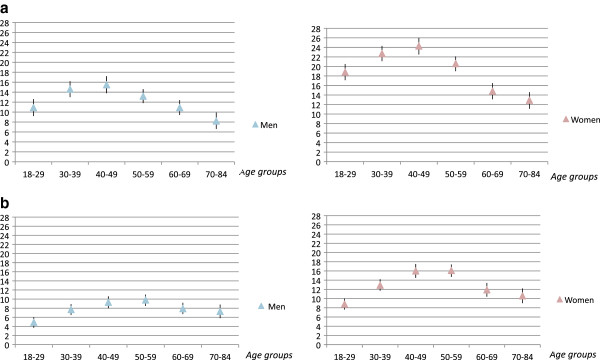
**a. The prevalence of short-duration BNP with corresponding 95% confidence intervals among men and women respectively.b.The prevalence of long-duration BNP with corresponding 95% confidence intervals among men and women respectively.**

The one-year prevalence of short- and/or long-duration BNP in 2006 was 25% (95% CI: 24–25) among women, and 16% (95% CI: 15–16) among men (Table 3). The prevalence peaked among individuals aged 30–59 years. The one-year prevalence of short-duration BNP was 20% (95% CI: 19–21) among women, and 13% (95% CI: 12–13) among men. The one-year prevalence of long-duration BNP was lower; 13% (95% CI: 13–14) among women, and 8% (95% CI: 8–9) among men. Figures 2a-2b and Table 3 show that short-and long-duration BNP was most common in the 30–49 and 40–59 age groups respectively.

**Table 3:** The age- and sex-specific one-year prevalence (%) with 95% confidence intervals (95% CI) of episodes of short- and/or long-duration BNP in 2006

**Age groups**	**All**	**18-29 years**	**30-39 years**	**40-49 years**	**50-59 years**	**60-69 years**	**70-84 years**
	**%**	**%**	**%**	**%**	**%**	**%**	**%**
	**(95% CI )**	**(95% CI)**	**(95% CI)**	**(95% CI)**	**(95% CI)**	**(95% CI)**	**(95% CI)**
	***n/N***^***a***^	***n/N***^***a***^	***n/N***^***a***^	***n/N***^***a***^	***n/N***^***a***^	***n/N***^***a***^	***n/N***^***a***^
**The prevalence of short-duration BNP**							
**Men**	**13**	**11**	**15**	**16**	**13**	**11**	**8**
	(12–13)	(9–13)	(13–16)	(14–17)	(12–15)	(9–12)	(7–10)
	*1,274/10,083*	*146/1,339*	*276/1,888*	*281/1,812*	*290/2,199*	*191/1,752*	*90/1,093*
**Women**	**20**	**19**	**23**	**24**	**21**	**15**	**13**
	(19–21)	(17–21)	(21–24)	(23–26)	(19–22)	(13–17)	(11–15)
	*2,529/12,765*	*372/1,979*	*619/2,828*	*559/2,305*	*547/2,658*	*258/1,742*	*174/1,353*
**The prevalence of long-duration BNP**							
**Men**	**8**	**5**	**8**	**9**	**10**	**8**	**7**
	(8–9)	(4–6)	(7–9)	(8–11)	(9–11)	(7–9)	(6–9)
	*830/10,298*	*66/1,356*	*147/1,918*	*172/1,849*	*221/2,266*	*141/1,776*	*83/1,133*
**Women**	**13**	**9**	**13**	**16**	**16**	**12**	**11**
	(13–14 )	(8–10)	(12–14)	(15–18)	(15–17)	(10–13)	(9–12)
	*1,725/13,146*	*176/2,001*	*362/2,803*	*381/2,380*	*442/2,753*	*213/1,790*	*151/1,419*
**The prevalence of short- and/or long-duration BNP**							
**Men**	**16**	**13**	**18**	**19**	**16**	**13**	**11**
	(15–16)	(12–15)	(16–19)	(17–21)	(15–18)	(12–15)	(9–12)
	*1,570/10,123*	*180/1,341*	335/1,896	346/1,821	365/2,220	229/1,749	115/1,096
**Women**	**25**	**22**	**28**	**30**	**27**	**19**	**16**
	(24–25)	(20–24)	(26–30)	(28–31)	(25–29)	(17–21)	(14–18)
	*3,164/12,878*	*440/1,981*	*768/2,745*	*688/2,328*	*717/2,704*	*330/1,760*	*221/1,360*

^a^n/N = number of neck pain cases/number of persons in the population.

The one-year incidence proportion of getting short- and/or long-duration BNP was 7% (95% CI: 6–7) among women and 4% (95% CI: 4–5) among men (Table 4). These proportions correspond to an incidence rate of 44 per 1,000 person-years among men, and 68 per 1,000 person-years among women, assuming one episode per individual and that each person contributed with one year of time at risk to get such pain. The incidence proportion was highest among both men and women under the age of 50.

**Table 4:** The age-specific one-year incidence proportion (%) with 95% confidence intervals (95% CI) of short- and/or long-duration BNP in 2006 among men and women without BNP in 2005, and of recovery from BNP in 2006 among men and women with short- and/or long-duration BNP in 2005

**Age groups**	**All**	**18-29 years**	**30-39 years**	**40-49 years**	**50-59 years**	**60-69 years**	**70-84 years**
	**%**	**%**	**%**	**%**	**%**	**%**	**%**
	**(95% CI)**	**(95% CI)**	**(95% CI)**	**(95% CI)**	**(95% CI)**	**(95% CI)**	**(95% CI)**
	**n/N**^***a***^	**n/N**^***a***^	**n/N**^***a***^	**n/N**^***a***^	**n/N**^***a***^	**n/N**^***a***^	**n/N**^***a***^
**The one-year incidence proportion of short- and/or long-duration BNP**							
**Men**	**4**	**6**	**5**	**5**	**4**	**3**	**3**
	(4–5)	(5–8)	(4–7)	(4–6)	(3–5)	(2–4)	(2–4)
	*384/8,747*	*75/1,211*	*86/1,601*	*77/1,500*	*73/1,893*	*47/1,545*	*26/997*
**Women**	**7**	**8**	**10**	**8**	**5**	**3**	**5**
	(6–7)	(7–10)	(8–11)	(7–10)	(4–6)	(2–4)	(3–6)
	*690/10,105*	*135/1,632*	*201/2,086*	*142/1,714*	*110/2,048*	*49/1,449*	*53/1,176*
**The one-year incidence proportion of recovery from short- and/or long-duration BNP**							
**Men**	**14**	**20**	**16**	**16**	**11**	**11**	**10**
	(12–16)	(13–27)	(12–20)	(12–21)	(7–14)	(7–15)	(4–17)
	*190/1,358*	*25/128*	*46/291*	*52/317*	*35/323*	*22/203*	*10/96*
**Women**	**11**	**13**	**14**	**11**	**8**	**10**	**9**
	(10–12)	(9–16)	(11–17)	(9–14)	(6–10)	(7–13)	(5–14)
	*299/2,728*	*44/344*	*92/652*	*68/605*	*49/651*	*30/301*	*16/175*

^*a*^n/N = number of neck pain cases/number of persons in the population.

The incidence proportion of recovery (at least one year of no BNP) was 11% (95% CI:10–12) among women and 14% (95% CI:12–16) among men (Table 4). The incidence proportion of recovery was highest in the 30–39 age group among women and in the 18–29 age group among men. Men recovered from BNP more frequently than women, at least in the younger age groups.

## Discussion

We found that bothersome neck pain (BNP) is common in the general population and therefore is an significant public health problem. Women were more likely than men to have and to develop BNP, and less likely to recover from such pain. This indicates that female gender is a risk factor as well as a negative prognostic factor and that the sex differences in prevalence are driven by differences in incidence as well as in recovery.

The prevalence as well as the incidence proportion and prognosis of BNP differ between age groups. The prevalence was highest in middle age, the incidence proportion was highest under the age of 50, and the incidence proportion of recovery was most favourable for women under the age of 40 and men under the age of 50. Accordingly, younger age seems to be a risk factor for BNP but also a prognostic factor for better recovery from such pain.

This is, to our knowledge, the first study to report on the age- and sex-specific prevalence and the incidence proportion of the onset and of the recovery from NP that is bothersome, from a large population-based sample. This public health problem probably makes a significant contribution to the global burden of disease, especially in middle-aged women. It is important to study prognostic factors as well as the underlying causal mechanism for this sex discrepancy in future studies.

### Comparison to other literature

In a systematic review, the one-year prevalence of activity-limiting NP in the general population was 1.7%-11.5% [2] referring to one survey among Hong Kong residents [17], and one survey among patients from British general practices [18]. This is lower than in our study and might be explained by a higher prevalence in Stockholm County [19], or by the fact that the definition of NP and recall periods differs across the surveys. Another explanation might be differences in response rate in different surveys. When comparing our results to other Swedish surveys [5,12] and a Danish survey [7], our results regarding prevalence are similar.

The oldest individuals in our cohort (70–84 years) had a one-year prevalence of short-and/or long-duration BNP of 16% among women and 11% among men, clearly lower than in the younger age groups. In Denmark, a higher one-month prevalence among 100-year-olds was found; 23% and 19% for women and men respectively [6]. This difference might be due to a more stringent definition of NP in our survey or that the prevalence is higher in the oldest age group, which is not included in our study. Differences in recall periods in the surveys might be another explanation. In another study, getting older did not increase the NP burden even though pain was found to persist longer in older groups [7]. Neither short- nor long-duration BNP was more common among the elderly than among other age groups in our study.

According to a recently published review, the one-year incidence of NP ranges between 10 and 21% [1]. The one-year incidence in our study was lower (4% for men and 7% for women), reflecting the more important NP surveyed in our study. By assuming that all individuals in the cohort free from BNP in 2005 contributed one year at risk to develop BNP in 2006, and that those reporting NP in 2006 had only one episode each, we calculated the incidence rate of BNP to be 44 per 1,000 person-years among men and 68 per 1,000 person-years among women. According to a systematic review, the incidence rate per 1,000 person-years ranges from 0.055 (for disc herniation with radiculopathy) to 179 (for self-reported NP) [2].

Our results indicate that younger age predicts a better course for men as well as for women. We have not found sex-specific estimates of the prognostic effect of age in the literature, but in the general population as a whole, younger age was reported to be prognostic of better recovery [11].

The evidence for the prognostic role of sex in NP outcome varies across studies [11]. We found that the prognosis was worse for women than for men.

### Methodological discussions

Our case definitions included information on four of the five axes in the classification proposed by the Bone and Joint Decade 2000–2010 Task Force on Neck Pain for enhancing comparisons between studies [13]. Our information was survey-based (Axis I) and referred to the general population (Axis II). We had some information on severity (Axis III) and we had full information on duration of the episodes (Axis IV). Like many other studies, we lacked information on each individual’s pattern of NP (Axis V), that is, we could not differentiate between single episodes, recurrent, and persistent pain. Accordingly we could not base our calculations of incidence proportions on those free of pain at baseline, but had to use the more demanding criterion of study participants being free from pain during the entire preceding year. Most probably this implies an underestimation of the true incidence proportion. In our opinion, the survey questions allowed us to arrive at a fairly reasonable estimate of the one-year period prevalence, although they do not formally meet the textbook definition of period prevalence (i.e. the sum of the number of cases prevalent at a point in time (prevalence) plus the number that occurs in a subsequent period (e.g. the subsequent year) [16].

The lack of information on the true number of episodes and their specific durations in our study also implies that only very crude estimates of incidence and recovery rate may be reported. However, the pattern of NP over time is usually not measured in detail in surveys, due to economical and practical reasons. We had to define recovery as at least one-year duration of no BNP, which is an arbitrary definition that may be uninformative especially if an individual case of BNP is recurrent with longer time intervals.

Crombie et al. suggest that a pain condition, such as NP is to be regarded as a dynamic process, since pain is characterized more by change than by stability [20], and that transitions between different NP states and changes in pain intensity and severity are to be expected. These limitations are the same as in most other studies in this area. We strongly believe that there is room for improvements in the musculoskeletal epidemiological research area, not only regarding the case definitions, but also regarding more details in the reporting of prevalence and incidence.

### Strengths and limitations

A major strength of this study is the large population-based sample, enabling analyses in subgroups of age and sex. In addition, we study NP that is bothersome, which is of high importance for the individual as well as for the society.

The main threat to the validity of our findings is the risk of bias in the one- to two-year retrospective recall of BNP from 2007 to 2006 and 2005 resulting in a misclassification of disease. Individuals with long-duration BNP might have overestimated the severity of prior pain periods, and individuals with recurrent pain might have forgotten about the severity of the pain in earlier episodes. The design of the cohort is prospective but since the follow-up time was five years, we used information about BNP from the follow-up questionnaire to report on the occurrence. The most valid information on BNP was judged to be from 2006 since that was the last full year before the questionnaire was answered in 2007. Nevertheless, additional analyses showed that the prevalence of BNP was two to three percentages points lower in 2005 than in 2006, indicating an underestimation of the prevalence that may have biased estimates of incidence proportion of getting BNP (overestimation) and incidence proportion of recovery (underestimation) between 2005 and 2006.

Another threat to the validity is the single generic question about BNP used on the questionnaire. It is probable that some individuals did not read the question carefully enough, and therefore have reported NP of less magnitude. This might overestimate the burden of BNP. Further the question used to define BNP are not validated to capture important neck pain that relates to disability, treatment requirements, sick leave and all associated disability, etc., even though the question has been shown to be valid for capturing important low back pain [15]. If it is not valid for BNP, our results might be biased and the neck pain reported on might be less important than stated.

The study population constitutes 48% of the 49,914 persons asked to take part in the baseline survey, and there were some internal dropouts, which threatens the external validity of the results. Since a higher proportion of the non-respondents were men under the age of 45, our results may be biased with a probable overestimation of the overall occurrence of BNP. The analyses of the smallest subgroups (e.g. in the youngest and oldest) were somewhat hampered by the lack of statistical power and should hence be interpreted with caution.

In summary we consider our results to be valid and that the study provides important information about age and sex differences relating to prevalence, incidence proportion and prognosis of BNP.

## Conclusion

In conclusion our results suggest that BNP is common among men and women and most common in middle-aged individuals. Women are more likely than men to have and to develop BNP, and less likely to recover from such pain. Younger men and women have a higher incidence, but they more often recover from BNP than older individuals.

## Abbreviations

NP: Neck pain; BNP: Bothersome neck pain; CI: Confidence interval.

## Competing interests

The authors declare that they have no competing interests.

## Authors' contributions

All authors contributed to the design of the study and interpreted data. ML performed the statistical analyses. ES wrote the first version of the manuscript. All authors critically revised different versions of the manuscript. All authors read the final version of the manuscript.

## Pre-publication history

The pre-publication history for this paper can be accessed here:

http://www.biomedcentral.com/1471-2474/13/185/prepub
